# ROS play an important role in ATPR inducing differentiation and inhibiting proliferation of leukemia cells by regulating the PTEN/PI3K/AKT signaling pathway

**DOI:** 10.1186/s40659-019-0232-9

**Published:** 2019-05-03

**Authors:** Yubin Feng, Xiaoxiao Hua, Ruowen Niu, Yan Du, Congjian Shi, Renpeng Zhou, Fei-Hu Chen

**Affiliations:** 1 Anhui Province Key Laboratory of Major Autoimmune Diseases, Anhui Institute of Innovative Drugs, School of Pharmacy, Anhui Medical University, Mei Shan Road, Hefei, 230032 Anhui China; 2 The Key Laboratory of Anti-inflammatory and Immune Medicines, Ministry of Education, Hefei, China

**Keywords:** Acute myeloid leukemia, Differentiation, Proliferation, ATPR, ROS, PTEN/PI3K/AKT

## Abstract

**Background:**

Acute myeloid leukemia (AML) is an aggressive and mostly incurable hematological malignancy with frequent relapses after an initial response to standard chemotherapy. Therefore, novel therapies are urgently required to improve AML clinical outcomes. 4-Amino-2-trifluoromethyl-phenyl retinate (ATPR), a novel all-trans retinoic acid (ATRA) derivative designed and synthesized by our team, has been proven to show biological anti-tumor characteristics in our previous studies. However, its potential effect on leukemia remains unknown. The present research aims to investigate the underlying mechanism of treating leukemia with ATPR in vitro.

**Methods:**

In this study, the AML cell lines NB4 and THP-1 were treated with ATPR. Cell proliferation was analyzed by the CCK-8 assay. Flow cytometry was used to measure the cell cycle distribution and cell differentiation. The expression levels of cell cycle and differentiation-related proteins were detected by western blotting and immunofluorescence staining. The NBT reduction assay was used to detect cell differentiation.

**Results:**

ATPR inhibited cell proliferation, induced cell differentiation and arrested the cell cycle at the G0/G1 phase. Moreover, ATPR treatment induced a time-dependent release of reactive oxygen species (ROS). Additionally, the PTEN/PI3K/Akt pathway was downregulated 24 h after ATPR treatment, which might account for the anti-AML effects of ATPR that result from the ROS-mediated regulation of the PTEN/PI3K/AKT signaling pathway.

**Conclusions:**

Our observations could help to develop new drugs targeting the ROS/PTEN/PI3K/Akt pathway for the treatment of AML.

## Introduction

Acute myeloid leukemia (AML) is a heterogeneous disease that affects 3–4 out of every 100,000 people, and the median age of AML patients is 67 years. The 5-year survival rate is approximately 20% [1]. The progression of the disease depends on many factors, including cytogenetics, molecular genetics, comorbidity scores, and the age of the patient. As the understanding of AML pathogenesis has increased cytotoxic chemotherapy with or without subsequent hematopoietic cell transplantation has been established as the primary treatment for AML. Despite many efforts to identify treatments for AML, the prognosis has not improved significantly over the past decade, and this endeavor remains a challenge [2]. Acute promyelocytic leukemia (APL) is a subtype of acute myeloid leukemia (AML) characterized by the accumulation of immature promyelocytes in the peripheral blood and the bone marrow. For decades, APL has been considered the most malignant AML because of the occurrence of severe bleeding in the disease and its high early mortality rate [3, 4]. Currently, retinoic acid (RA) and arsenic trioxide (ATO) are two classic drugs used for the treatment of APL. Treatments for APL are associated with a number of issues, such as ATO or all-trans retinoic acid (ATRA) resistance, relapse, differentiation syndrome and adverse effects [5–8]. In addition, ATRA seems to be a poor treatment for non-APL. Therefore, it is necessary to identify other therapeutic strategies for AML, including APL (using NB4 cells) and non-AML (using THP-1 cells).

**Fig. 1:**
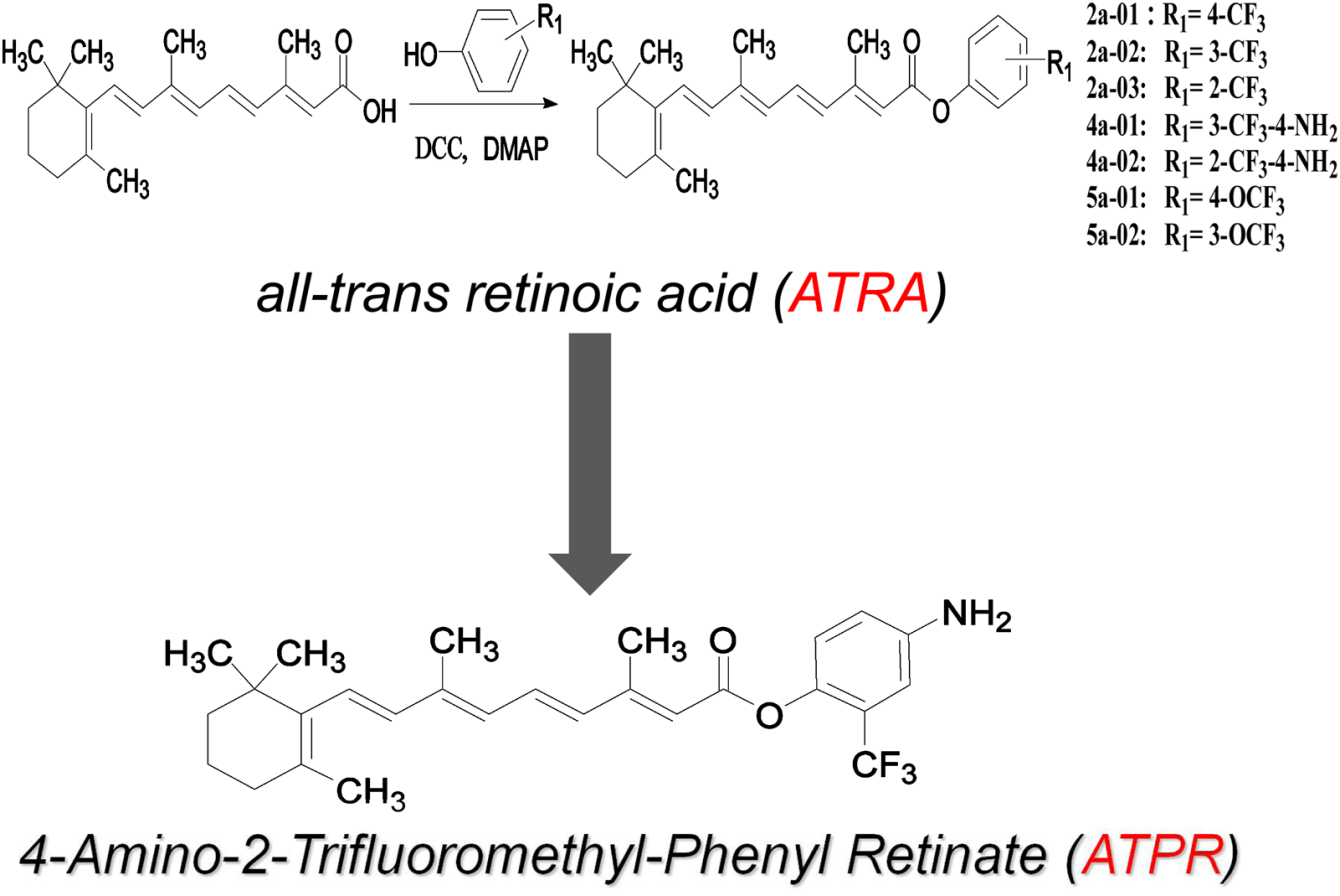
Synthesis of ATPR by structural modification of ATRA

To overcome the side effects of ATRA, our team has altered the structure of ATRA to obtain a series of retinoic acid derivatives. After pre-pharmacodynamic screening, we found that 4-amino-2-trifluoromethyl-phenyl retinate (ATPR) (Fig. 1) has a favorable anti-tumor effect. ATPR shows better solubility than ATRA [9]. The anti-tumor effect has been studied in several types of solid tumors. Some studies have shown that ATPR can effectively inhibit growth and differentiation induction in breast cancer MCF-7 cells and gastric cancer SGC-7901 cells via the upregulation of retinoid receptor-induced gene-1 or retinoic acid receptors [10, 11]. These studies suggest that ATPR can exhibit strong anti-tumor effects and has potential as a cancer chemotherapeutic agent, but the molecular mechanism remains unclear.

Reactive oxygen species (ROS) are primarily produced by NADPH oxidase (Nox), an important cellular signaling molecule involved in the progression of cancer cells, and are generally thought to be second messengers that augment inflammation by activating downstream signal cascades [12, 13]. Phosphatase and tensin homolog (PTEN) plays an important role in mature organisms as a tumor suppressor. The inactivation of PTEN genes by mutation or deletion is common in pediatric T-cell acute lymphoblastic leukemia (T-ALL) [14]. The major substrate of PTEN is phosphatidylinositol-3,4,5-triphosphate (PIP3), which is produced by the action of phosphoinositide-3-kinase (PI3K) [15]. The PI3K/AKT signaling pathway plays an important role in the development of anticancer therapies, and the inhibition of the PI3K/AKT signaling pathway may induce cycle arrest and differentiation in vitro.

Our results demonstrated that ATPR, a novel derivative of ATRA, inhibits the proliferation and induces the differentiation of acute myelocytic leukemia cells via the ROS-mediated regulation of the PTEN/PI3K/Akt signaling pathway. These findings suggest that ATPR may be a promising agent for acute myelocytic leukemia treatment.

## Materials and methods

### Chemicals and reagents

ATPR (purity: 99.66%) was synthesized by our laboratory (School of Pharmacy, Anhui Medical University). A 10^−2^ mol/l stock solution of ATPR was prepared in absolute alcohol and stored at − 20 °C. In addition, no effect of the solvent (alcohol) was found. Antibodies against cyclin A2, cyclin D3, CDK4, Rb (phosphatase), PTEN, AKT, phospho-Akt (Ser473), PI3K p85, CD11b (PE/CY5-conjugated anti-human CD11b) and CD14 (FITC-conjugated anti-human CD14) were obtained from Abcam (Danvers, MA, USA). β-Actin antibodies were purchased from Bioss. All of the antibodies used in the WB assay were diluted to 1:1000.

### Cell lines and cell culture

The human leukemia cell lines NB4 and THP-1 were purchased from Gecko Gene (Shanghai, China). They were cultured in RPMI 1640 medium supplemented with 10% fetal bovine serum (FBS, Gibco, USA), 100 U/ml penicillin and 100 μg/ml streptomycin in a 37 °C, 5% CO_2_ incubator. The medium was changed every 2 or 3 days. Cells in exponential growth were subjected to the following experiments.

### Cell viability assay

The indicated concentrations of ATPR (0, 10^−9^, 10^−8^, 10^−7^, 10^−6^, or 10^−5^ mol/l) were used to treat the cells for 24, 48, and 72 h. After the addition of the Cell Counting Kit-8 (CCK-8) solution (10 μl per well), the cells were then incubated at 37 °C for 1 h. The optical density (OD) levels were measured at 450 nm using a BioTek ELx808 Microplate Reader. The results are presented as the viability rates. Cell viability was calculated according to the following formula: cell viability (%) = (OD treatment − OD blank)/(OD control − OD blank).

### Cell cycle analysis

NB4 and THP-1 cells were seeded in six-well plates at a density of 2 × 10^5^ cells per well. Then, the cells were treated with ATPR (10^−6^ mol/l) for 24 h, 48 h and 72 h at 37 °C. For cell cycle analysis, the cells were harvested, washed twice with PBS and fixed in 70% ethanol at 4 °C overnight. After a 15-min incubation period with 20 μl RNase A plus 400 μl propidium iodide (PI), the cells were subjected to cell cycle analysis using CytoFLEX (Becton–Dickinson, USA). The cell cycle distribution was analyzed by ModFIT software (BD Biosciences).

### Differentiation marker analysis

Then, the cells were treated with ATPR (10^−6^ mol/l) for 24 h, 48 h and 72 h at 37 °C. After treatment, the cells were washed with PBS and then incubated with monoclonal antibodies against CD11b (CD11b-PE/CY5) and CD14 (CD14-FITC) for 30 min on ice away from light. The cells were then washed twice with ice-cold PBS and finally resuspended in 500 μl PBS for measurement. CD11b and CD14 expression levels were measured using CytoFLEX (Becton–Dickinson, USA). The data were processed using CytoFLEX.

### Nitroblue tetrazolium (NBT) assay

For the NBT reduction analysis, NB4 and THP-1 cells (1 × 10^5^ cells/ml) were seeded in a 6-well plate and treated with ATPR. A 10-μl aliquot of the NBT solution, composed of 10 mg/ml NBT (Sigma) and 2 μg/ml PMA (Sigma), was added to each well. Then, the cells were incubated for 30 min at 37 °C. Both the percentage of positive cells and the intensity of reduction when measured at a wavelength of 570 nm with a BioTek ELx808 Microplate Reader was evaluated in the NBT test.

### Measurement of intracellular ROS

Intracellular ROS were analyzed by 2′-7′-dichlorofluorescein diacetate (DCFH-DA; Beyotime Biotechnology). After treatment, the cells were washed with PBS and incubated with 20 μM DCFH-DA diluted in RPMI 1640 medium for 30 min at 37 °C in the dark. The cells were then washed with PBS and analyzed using CytoFLEX (Becton–Dickinson, USA). For the flow cytometry assay, the cells were analyzed using CytoFLEX. The ROS inhibitors NAC and Tiron were purchased from TagerMol.

### Western blot analysis

NB4 and THP-1 cells were washed twice with prechilled PBS after treatment with different concentrations of ATPR (0, 10^−9^, 10^−8^, 10^−7^, 10^−6^, or 10^−5^ mol/l) for 72 h or with 10^−6^ mol/l ATPR at different time points (24, 48, and 72 h). Total protein was extracted using RIPA lysis buffer plus a protease inhibitor cocktail and then quantified by a BCA Protein Assay Kit (Beyotime). Equal amounts of protein (30 μg/well) were subjected to 10% or 12% SDS-PAGE and then electrotransferred onto 0.45 μm PVDF membranes (Millipore Corp). The membranes were blocked with 5% nonfat milk or bovine serum albumin for 2 h at room temperature followed by overnight incubation at 4 °C in primary antibodies as described above. After washing with TBST, the membranes were incubated with the indicated HRP-conjugated secondary antibodies for 1 h at room temperature. The blots were visualized with an ECL kit (ECL-plus; Thermo Fisher Scientific). ImageJ software was used to quantify the band intensity. For each sample, the signal intensity for each protein was normalized to that of β-actin and is expressed as the fold-increase relative to the control.

### Immunofluorescence staining

NB4 and THP-1 cells were plated on 35-mm glass-bottom culture dishes. After 24 h and 72 h of treatment with ATPR (10^−6^ mol/l), the cells were fixed with 4% paraformaldehyde for 15 min. The fixed cells were permeabilized with 0.5% Triton X-100 for 10 min and blocked with 10% normal goat serum for 30 min. Then, the cells were incubated with a primary antibody against p-AKT (1:200) at 4 °C overnight. After rinsing with PBS three times for 5 min each time, the cells were incubated with a fluorescent-labeled secondary antibody in the dark for 1 h. Finally, the nuclei were counterstained with DAPI and examined and imaged using a NIKON fluorescence microscope.

### Statistical analysis

Each experiment was repeated at least three times, and the data are shown as the mean ± standard deviation (SD). Statistical analyses were performed using SPSS software version 20.0 (SPSS Inc., Chicago, IL, USA). Between-group differences were estimated with one-way analysis of variance (ANOVA). p < 0.05 was considered to be statistically significant. All experiments were repeated three times.

## Results

### ATPR inhibits cell proliferation in human leukemia cells

**Fig. 2:**
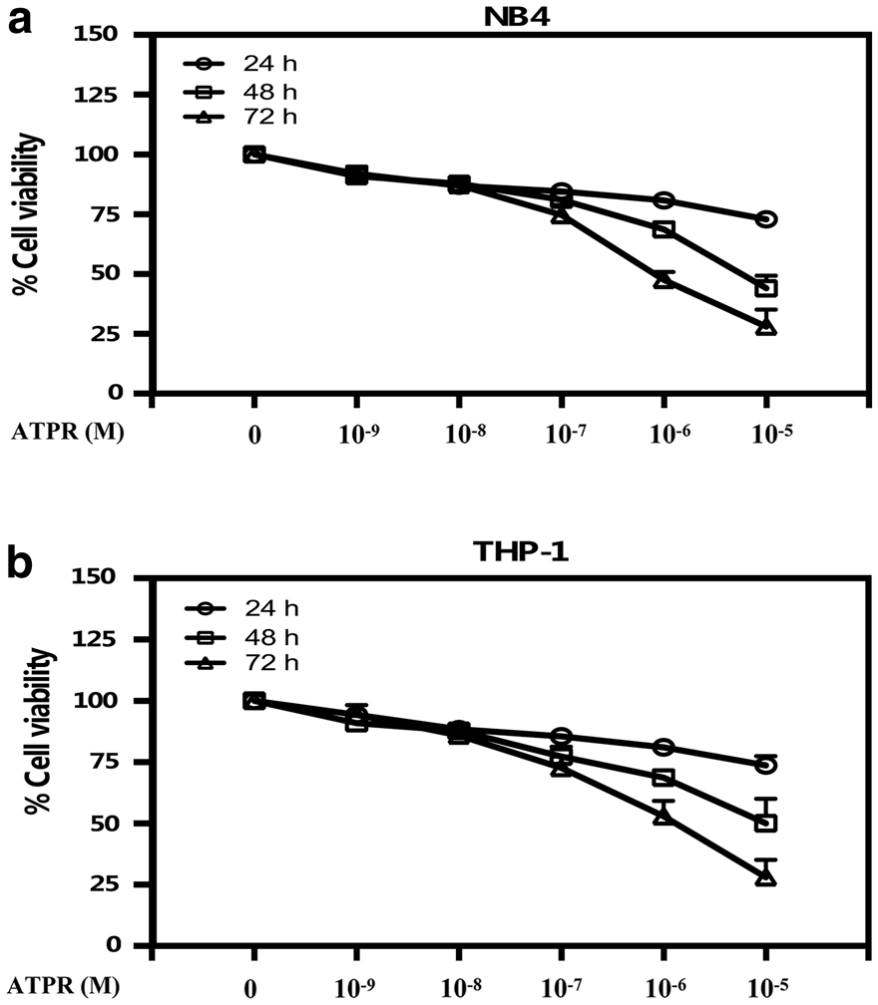
ATPR inhibits cell proliferation in human NB4 and THP-1 cells. **a**, **b** Dose- and time-dependent effects of ATPR on NB4 and THP-1 cells. NB4 and THP-1 cells were exposed to various concentrations (0, 10^−9^, 10^−8^, 10^−7^, 10^−6^, or 10^−5^ M) of ATPR for 24–72 h, followed by the determination of cell viability using the CCK8 assay. Values are presented as the mean ± SD of three independent experiments. *p < 0.05, **p < 0.01 versus control group

**Fig. 3:**
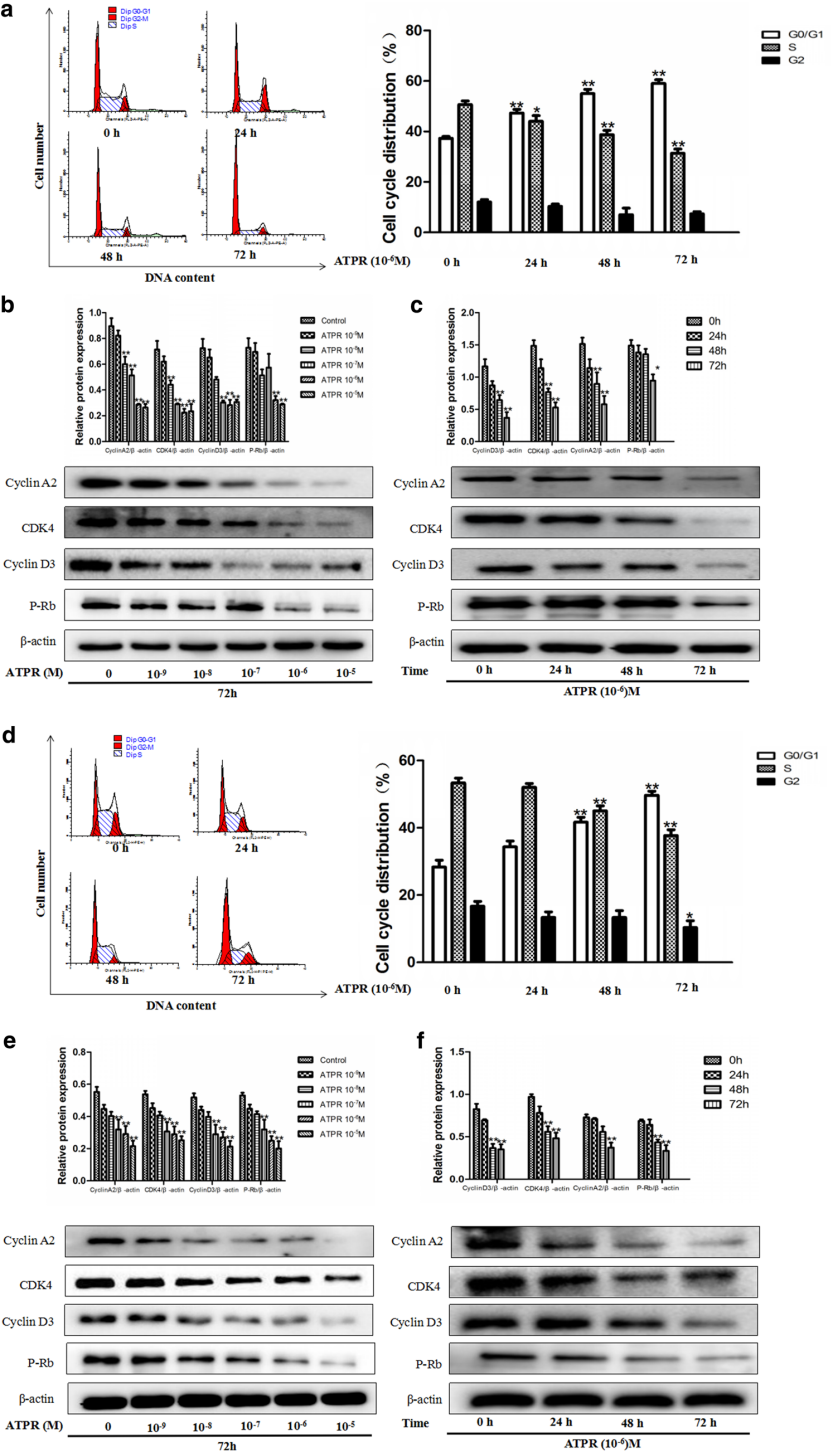
ATPR triggers cell cycle arrest at G0/G1 phase in human NB4 and THP-1 cells. **a** NB4 cells were treated with ATPR (10^−6^ M) for different durations (0–72 h). The cell cycle distribution was analyzed by flow cytometry. **b** NB4 cells were treated with an ATPR concentration gradient (10^−5^–10^−9^ M) for 72 h. Then, the protein expression of cyclin A2, CDK4, cyclin D3 and p-Rb was assessed by western blot. **c** NB4 cells were treated with ATPR (10^−6^ M) for different duration (0–72 h). Then, the protein expression of cyclin A2, CDK4, cyclin D3 and p-Rb was assessed by western blot. **d** THP-1 cells were treated with ATPR (10^−6^ M) for different durations (0–72 h). The cell cycle distribution was analyzed by flow cytometry. **e** THP-1 cells were treated with an ATPR concentration gradient (10^−5^–10^−9^ M) for 72 h. Then, the protein expression of cyclin A2, CDK4, cyclin D3 and p-Rb was assessed by western blot. **f** THP-1 cells were treated with ATPR (10^−6^ M) at different time points (0–72 h). Then, the protein expression of cyclin A2, CDK4, cyclin D3 and p-Rb was assessed by western blot. *p < 0.05, **p < 0.01 versus control group

In order to explore the proliferative effect of ATPR on AML cells, the CCK8 assay was used. As shown in Fig. 2a, b, ATPR inhibited the viability of NB4 and THP-1 cells in a time- and dose-dependent manner. The IC_50_ value of ATPR in NB4 and THP-1 cells at 24, 48, and 72 h was 10^−6^ M. As shown in Fig. 3a, d, a time-dependent accumulation of cells in G0/G1 phase was observed after ATPR treatment. However, the percentage of cells in S phase was reduced after ATPR treatment. These results indicate that NB4 and THP-1 cells were arrested in G0/G1 phase by ATPR. Moreover, Fig. 3b, c, e, and f shows that ATPR downregulated the expression of cyclin D3, cyclin A2, CDK4 and p-Rb, which are marker proteins of G0/G1 phase. These results also indicate that ATPR inhibited NB4 and THP-1 cell proliferation in a time- and concentration-dependent manner.

### ATPR induces differentiation in NB4 and THP-1 cells

**Fig. 4:**
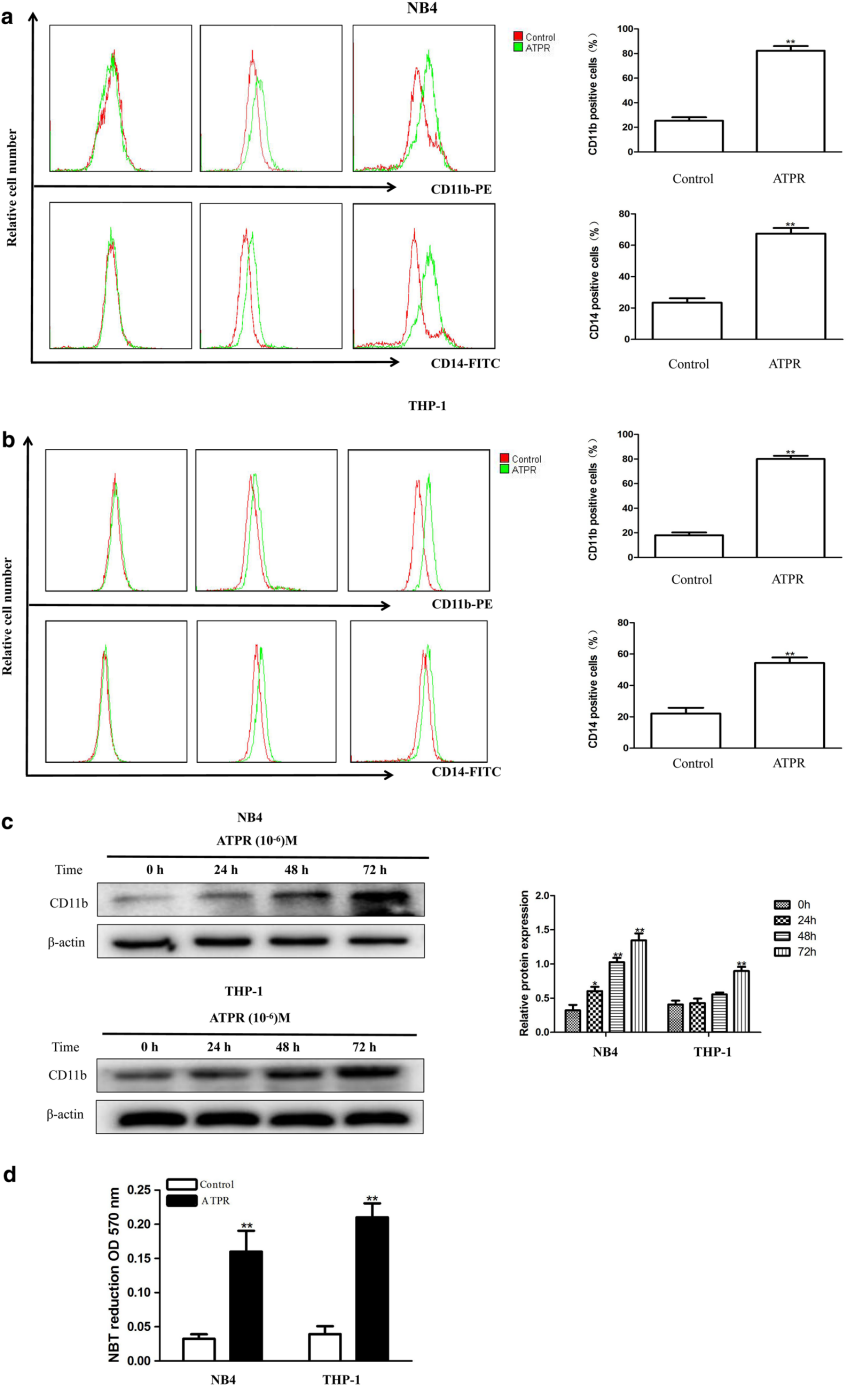
ATPR induces differentiation in human NB4 and THP-1 cells. **a** NB4 cells were treated with ATPR (10^−6^ M) for different durations (0–72 h). CD11b and CD14 expression was analyzed by flow cytometry. **b** THP-1 cells were treated with ATPR (10^−6^ M) for different durations (0–72 h). CD11b and CD1 expression was analyzed by flow cytometry. **c** NB4 and THP-1 cells were treated with ATPR (10^−6^ M) for different durations (0–72 h). Then, the protein expression of CD11b was assessed by western blot. **d** NB4 and THP-1 cells were treated with ATPR (10^−6^ M) for 72 h, and the NBT reduction assay detected cell differentiation. *p < 0.05, **p < 0.01 versus control group

To investigate whether ATPR induces differentiation in NB4 and THP-1 cells, we measured CD11b and CD14 surface antigen expression in the treated cells. Flow cytometry showed that ATPR remarkably increased CD14 and CD11b expression levels in a time-dependent manner (Fig. 4a, b). Furthermore, western blot analysis also showed that CD11b expression was remarkably higher in ATPR-treated cells than in the control group (Fig. 4c). Finally, we found that ATPR-treated cells exhibited increased nitroblue tetrazolium (NBT) reduction (Fig. 4d). Altogether, these findings strongly suggest that ATPR induced leukemia cell differentiation.

### ATPR can regulate the PTEN/PI3K/AKT signaling pathway

**Fig. 5:**
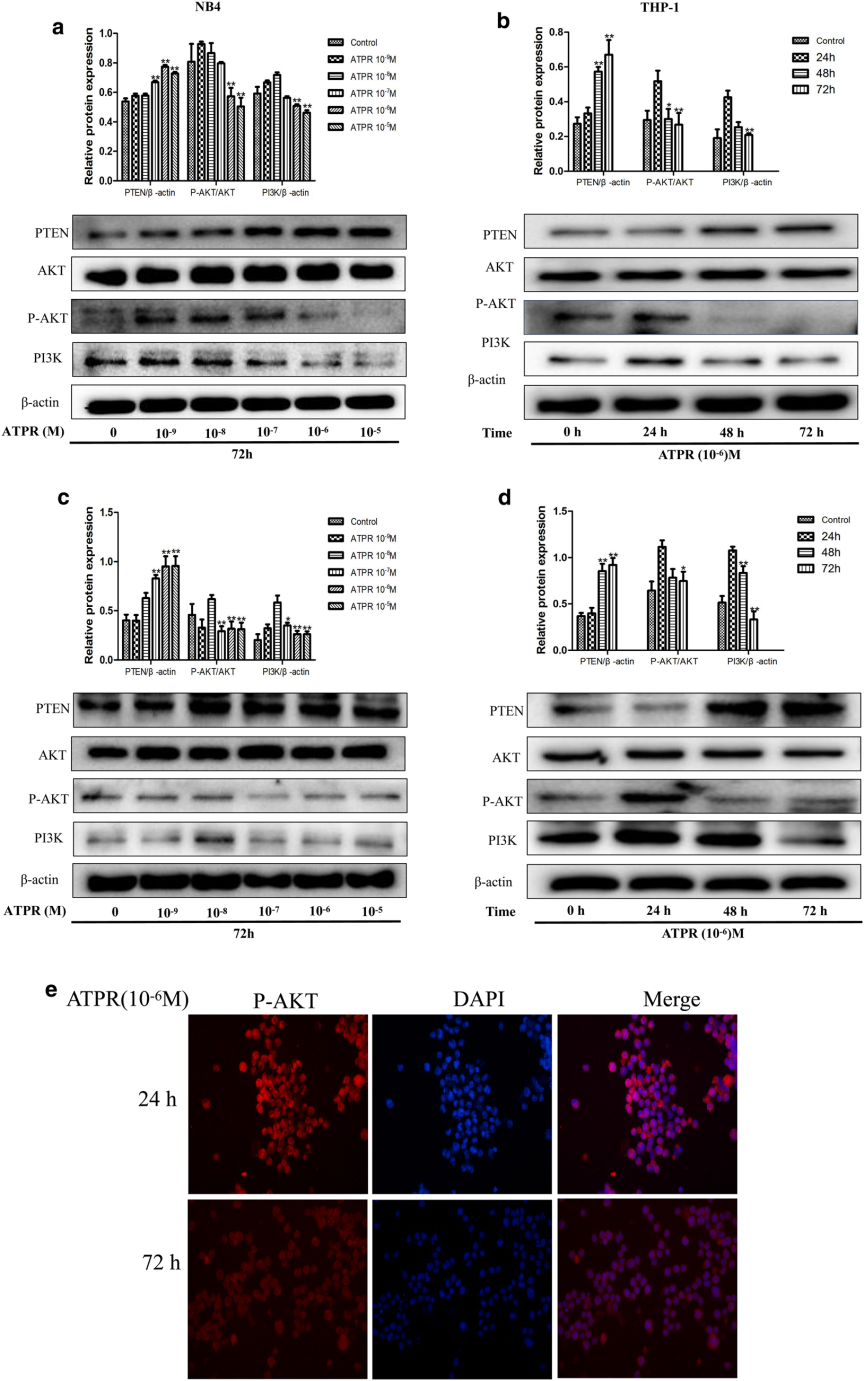
ATPR regulates the PTEN/PI3K/Akt pathway in human NB4 and THP-1 cells. **a** NB4 cells were treated with an ATPR concentration gradient (10^−5^–10^−9^ M) for 72 h. Then, the protein expression of PTEN, AKT, p-AKT and PI3K was assessed by western blot. **b** NB4 cells were treated with ATPR (10^−6^ M) for different durations (0–72 h). Then, the protein expression of PTEN, AKT, p-AKT and PI3K was assessed by western blot. **c** THP-1 cells were treated with an ATPR concentration gradient (10^−5^–10^−9^ M) for 72 h. Then, the protein expression of PTEN, AKT, p-AKT and PI3K was assessed by western blot. **d** THP-1 cells were treated with ATPR (10^−6^ M) for different durations (0–72 h). Then, the protein expression of PTEN, AKT, p-AKT and PI3K was assessed by western blot. **e** NB4 cells were treated with ATPR (10^−6^ M) for 24 h and 72 h. Immunofluorescence was used to detect p-Akt. *p < 0.05, **p < 0.01 versus control group

ATPR increased PTEN expression in a time- and concentration-dependent manner (Fig. 5a–d). Next, we assessed the changes in PI3K/AKT-related marker proteins, e.g., PI3K, AKT, and p-AKT. The cells were exposed to ATPR at a concentration of 10^−6^ M for the indicated time points (0, 24, 48 and 72 h). As shown in Fig. 5a, c, p-Akt (Ser473) and PI3K expression was induced when the cells were treated with 10^−8^ M ATPR and decreased upon exposure to increasing ATPR concentrations, up to 10^−5^ M. As shown in Fig. 5b, d. p-Akt (Ser473) and PI3K expression was induced when the cells were treated with ATPR for 24 h and decreased upon exposure to ATPR for increasing durations, up to 72 h. Furthermore, the decreased expression of p-Akt (Ser473) was confirmed both in the cytoplasm and nucleus by immunofluorescence staining after 24 h of treatment with ATPR (Fig. 5e). Therefore, our data demonstrate that ATPR regulates the PTEN/PI3K/Akt pathway in NB4 and THP-1 cells.

### ATPR promotes intracellular ROS accumulation, an important event in ATPR-induced cycle arrest and differentiation

**Fig. 6:**
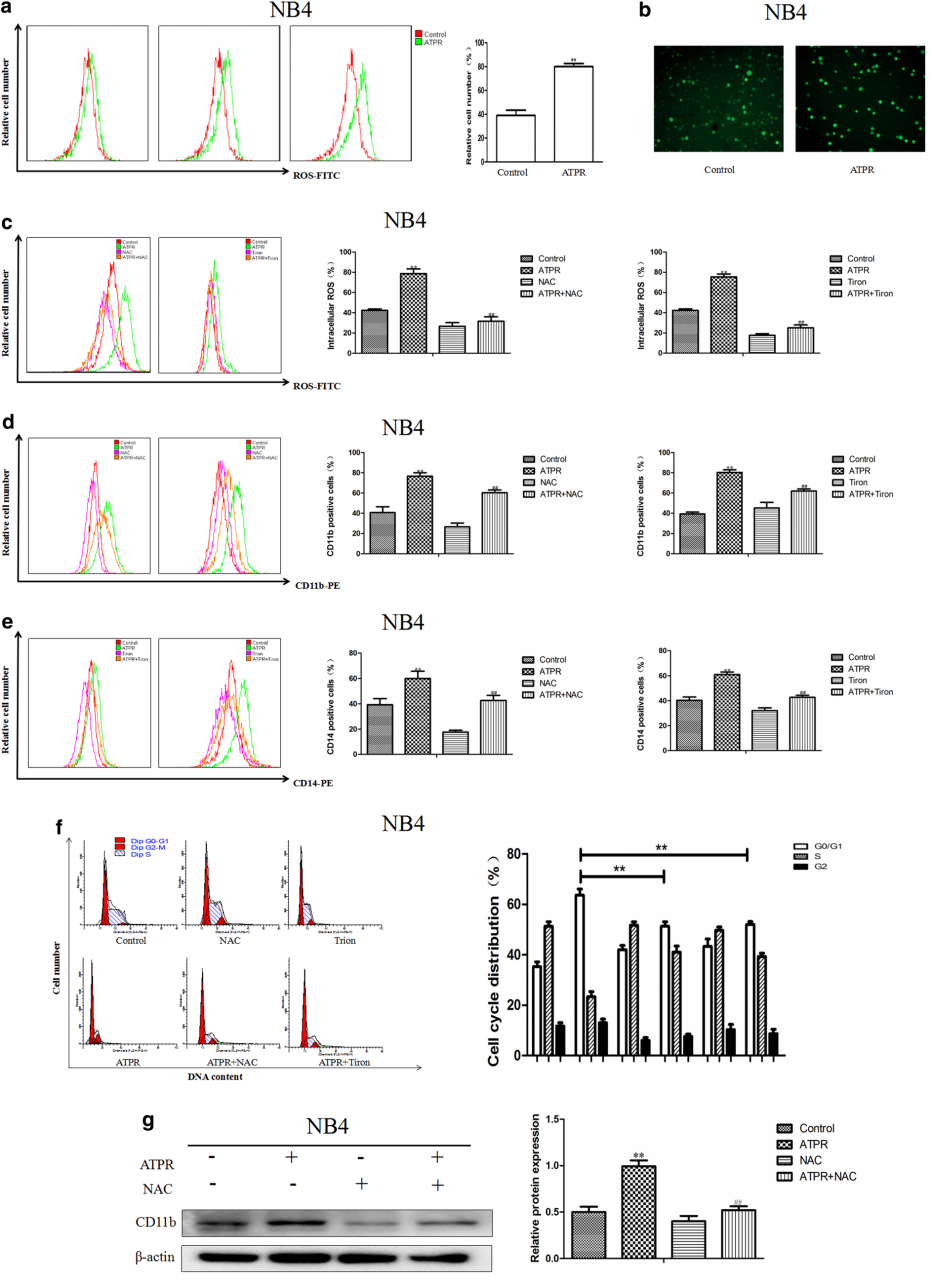
ATPR induces the accumulation of intracellular ROS, leading to cell cycle arrest at G0/G1 and differentiation. **a** NB4 cells were treated with ATPR (10^−6^ M) for different durations (0–72 h). Intracellular ROS levels were assessed by flow cytometry. **b** Intracellular ROS levels were assessed by immunofluorescence staining after treatment with 10^−6^ M ATPR for 72 h. **c**–**e** NB4 cells were pretreated with NAC and Tiron for 1 h and then treated with ATPR (10^−6^ M) for 72 h. The intracellular ROS levels were measured by flow cytometry, and CD11b and CD14 expression was measured by flow cytometry. **f** NB4 cells were pretreated with NAC for 1 h and then treated with ATPR (10^−6^ M) for 72 h. CD11b levels were analyzed by western blot. *p < 0.05, **p < 0.01 versus control group; ^#^p < 0.05, ^##^p < 0.01 versus ATPR group

As shown in Fig. 6a, b, we found that ATPR-treated NB4 cells had higher intracellular ROS levels than that of the control group cells. To further investigate whether ROS accumulation was involved in ATPR-induced cell cycle arrest and differentiation, CD11b and CD14 expression was assessed after ATPR treatment in the presence or absence of *N*-acetyl-l-cysteine (NAC) and Tiron. The results indicated that ATPR-induced ROS generation, cell cycle arrest and differentiation were markedly restrained by NAC and Tiron (Fig. 6c–f), and western blot analysis of CD11b expression revealed similar results (Fig. 6g).

### ROS can regulate the PTEN/PI3K/AKT signaling pathway

**Fig. 7:**
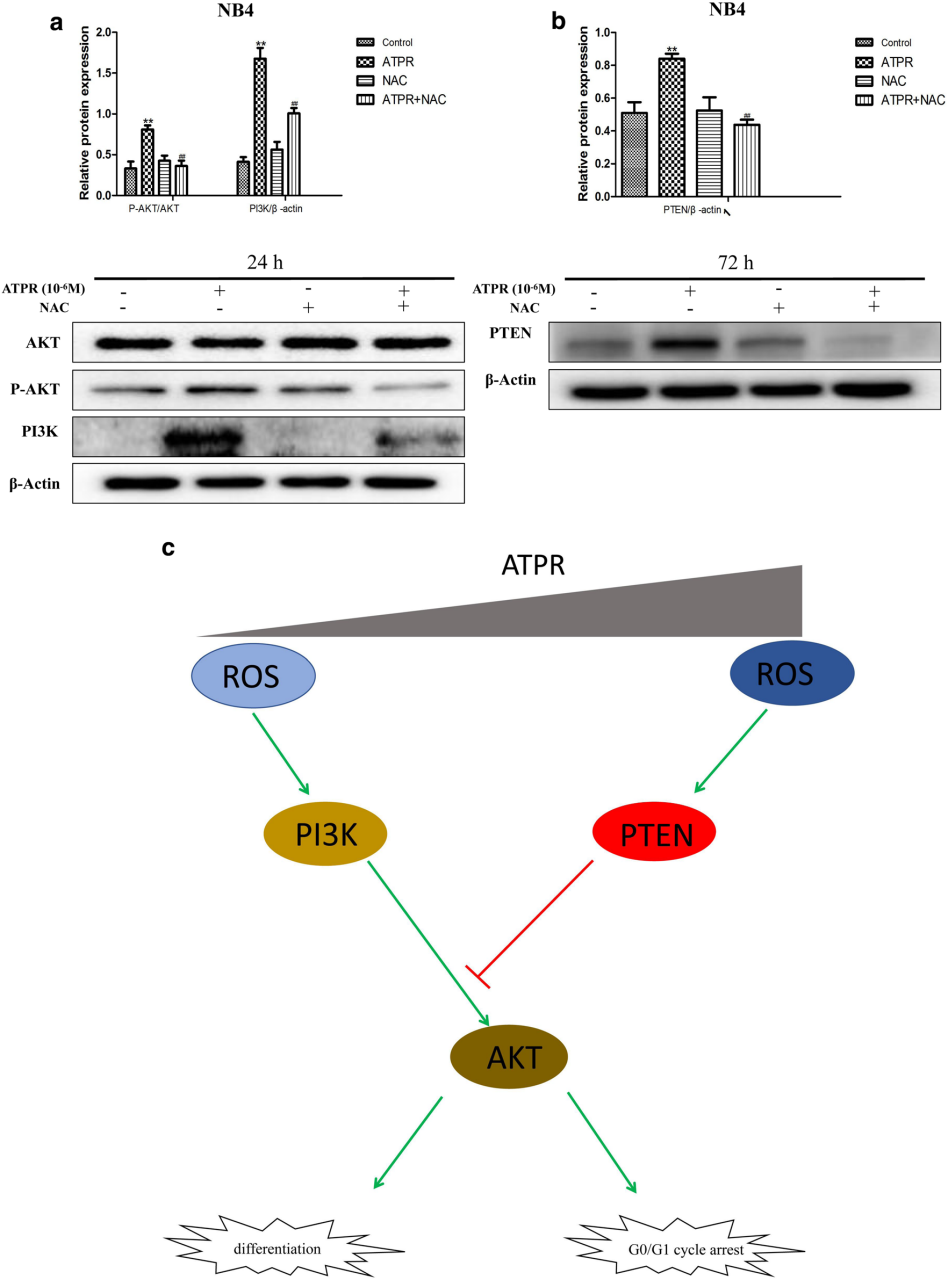
Assessment of PTEN/PI3K/AKT expression in NB4 cells after exposure to ATPR. **a** NB4 cells were pretreated with NAC for 1 h and then treated with ATPR (10^−6^ M) for 24 h. PI3K, AKT and P-AKT levels were analyzed by western blot. **b** NB4 cells were pretreated with NAC for 1 h and then treated with ATPR (10^−6^ M) for 72 h. **b** PTEN levels were analyzed by western blot. **c** A schematic of the proposed mechanism of ATPR-induced G0/G1 arrest and differentiation in human leukemia cells. The red arrows represent the inhibition of the subsequent signal, while the green arrows represent the promotion of the subsequent signal. *p < 0.05, **p < 0.01 versus control group; ^#^p < 0.05, ^##^p < 0.01 versus ATPR group

Similarly, NAC was used to examine the relationship between the PTEN/PI3K/AKT signaling pathway and the generation of ROS. Interestingly, Fig. 7a shows that NAC inhibited the activation of the PI3K/AKT signaling pathway. Furthermore, NAC inhibited the induction of PTEN expression (Fig. 7b). ROS may play an important role in the ATPR-mediated induction of differentiation and proliferation inhibition of leukemia cells by regulating the PTEN/PI3K/AKT signaling pathway (Fig. 7c).

## Discussion

A high recurrence rate, a high progression rate and high mortality are characteristics of acute myelocytic leukemia (AML). There is an urgent need to identify the potential molecular mechanisms of and to develop a new anti-tumor treatment for acute myelocytic leukemia. According to previous reports, ATPR is a potential drug for the treatment of specific cancers [4]. The CCK-8 assay data in Fig. 1 of this study demonstrate that ATPR inhibited the proliferation of the human acute myelocytic leukemia cell lines NB4 and THP-1 in a time- and dose-dependent manner. The cell cycle proceeds sequentially through four phases, including G0/G1, S, G2, and M. Cell cycle dysregulation is one of the most common alterations in tumor development [19]. Blocking the cell cycle is often considered an effective strategy to eliminate cancer cells [20]. The results of previous studies indicate that ATPR arrests gastric cancer cells at G0/G1 stage of the cell cycle [11]. Our data show that ATPR induced G0/G1 arrest in NB4 and THP-1 cells in a time-dependent manner (Fig. 3a, d). ATPR-induced G0/G1 arrest may help NB4 and THP-1 cells properly repair DNA defects, thus preventing their transmission to the resulting daughter cells. The transition of the cell cycle from one phase to the next requires a regulatory mechanism called checkpoints. CDK/cyclin complexes are often involved in these checkpoints [16, 17]. Meanwhile, our results also show that ATPR reduced the expression of the G0/G1 cycle marker proteins cyclin D3, CDK4, p-Rb and cyclin A2 (Fig. 3b, c, e, f). Cells at the G0/G1 checkpoint are surveyed to determine whether their DNA replicates correctly, and these cells will not transition to the next phase until replication problems are solved. If DNA repair does not occur after some time, cells will undergo differentiation. Figure 4 of this study also shows that ATPR induced cell cycle arrest and inhibited DNA synthesis, subsequently leading to differentiation in NB4 and THP-1 cells. Increasing evidence shows that the PI3K/Akt pathway is involved in the pathogenesis of many cancers and regulates a variety of cellular processes, including differentiation, proliferation, metastasis and metabolism [18]. Therefore, we wanted to explore whether the anti-leukemia effect of ATPR is related to this pathway.

PI3Ks are divided into three categories (I, II, and III), and class I PI3K includes two subgroups, IA and IB. The widely studied class IA PI3K, which plays a key role in tumorigenesis, is a heterodimer composed of the p85 regulatory subunit and the p110 catalytic subunit [19]. The activation of PI3K converts PI (3, 4) P2 to PI (3, 4, 5) P3, a process that can be reversed by PTEN. PIP3, as a second messenger, recruits Akt to the plasma membrane where Akt is phosphorylated at Thr308 and Ser473 [20]. Activated Akt may promote cell cycle progression and tumor growth through its downstream p53 pathway and Bcl-2 family proteins. Akt phosphorylates mouse double minute 2 (MDM2), an E3 ubiquitin ligase that triggers p53 degradation [21]. The tumor suppressor gene p53 induces cell cycle arrest and apoptosis by increasing the expression of its two transcriptional targets, p21^Waf1/Cip1^ and Bax, respectively [22]. The PI3K/Akt pathway is considered to play a major role in acute myelocytic leukemia, but the specific mechanisms of its role remain to be investigated [23]. PTEN is an essential tumor suppressor that antagonizes the PI3K/AKT anti-apoptotic pathway [24, 25]. Our study found that ATPR promoted the upregulation of PTEN in a time- and concentration-dependent manner. Theoretically, PI3K/AKT should be inhibited when PTEN levels are increased. However, during the treatment of AML by ATPR, the relevant target proteins of the PI3K/AKT signaling pathway first increase and then decrease. This is a very interesting finding that has attracted our attention. It has been reported that ROS play a regulatory role in the PTEN/PI3K/AKT signaling pathway [26, 27]. It remains to be determined whether ROS play an important role in the ATPR-induced differentiation and proliferation inhibition of leukemia cells by regulating the PTEN/PI3K/AKT signaling pathway.

We observed that ATPR treatment significantly stimulates ROS generation (Fig. 6a, b). We also observed that the ROS scavengers NAC and Tiron can inhibit ATPR-induced differentiation (Fig. 6d, e, g) and G0/G1 cycle arrest (Fig. 6f). These results suggest that ATPR promotes intracellular ROS production, which is very important for the anti-leukemia effects of ATPR in cells. Initially, ATPR activates PI3K/Akt pathway signaling, but ROS inhibitors can reverse this behavior (Fig. 7a). As the levels of reactive oxygen species increase, PTEN is activated (Fig. 7b), which in turn inhibits the PI3K/AKT signaling pathway (Fig. 7c).

To our knowledge, this is the first report to demonstrate the anti-neoplastic effect of ATPR on acute myelocytic leukemia via the ROS-mediated regulation of the PTEN/PI3K/AKT pathway. However, it is worth noting that ATPR has good solubility in anhydrous ethanol but poor solubility in water, which poses a serious obstacle to its practical application as a drug for leukemia treatment. Injectable ATPR is being developed and may contribute to the wider use of ATPR for future research. Further studies on the characteristics of acute myelocytic leukemia are also needed to better understand the therapeutic potential of ATPR.

## Conclusions

In summary, our observations show that ATPR inhibited the proliferation and induced the differentiation of AML cells and that this effect was related to the ROS-mediated regulation of the PTEN/PI3K/AKT signaling pathway. These studies provide a rationale for the application of ATPR in clinical therapies and in therapeutic regimens for AML patients.

## Abbreviations

AML: acute myeloid leukemia
ATPR: 4-amino-2-trifluoromethyl-phenyl retinate
ATRA: all-trans retinoic acid
PML-RARα: promyelocytic leukemia-retinoic acid receptor alpha
RA: retinoic acid
ATO: arsenic trioxide
ROS: reactive oxygen species
PTEN: phosphatases and tensin homolog
T-ALL: T cell acute lymphoblastic leukemia
PI3K: phosphoinositide-3-kinase
CCK-8: cell counting kit-8
FBS: fetal bovine serum
OD: optical density
RIPA: radio-immunoprecipitation assay
NBT: nitroblue tetrazolium
PVDF: polyvinylidene difluoride
SDS: sodium dodecyl sulfate
PAGE: polyacrylamide gel electrophoresis
DAPI: 2-(4-amidinophenyl)-6-indolecarbamidine dihydrochloride
